# DNA barcoding reveals diversity of Hymenoptera and the dominance of parasitoids in a sub-arctic environment

**DOI:** 10.1186/1472-6785-13-2

**Published:** 2013-01-26

**Authors:** Julie K Stahlhut, José Fernández-Triana, Sarah J Adamowicz, Matthias Buck, Henri Goulet, Paul DN Hebert, John T Huber, Mark T Merilo, Cory S Sheffield, Thomas Woodcock, M Alex Smith

**Affiliations:** 1 Biodiversity Institute of Ontario, University of Guelph, Guelph, ON, Canada; 2 School of Environmental Sciences, University of Guelph, Guelph, ON, Canada; 3 Natural Resources Canada, Ottawa, ON, Canada; 4 Canadian National Collection of Insects, Arachnids, and Nematodes, Agriculture and Agri-Food Canada, Ottawa, ON, Canada; 5 Royal Alberta Museum, Edmonton, AB, Canada; 6 Royal Saskatchewan Museum, Regina, SK, Canada

**Keywords:** Barcoding biotas, Biodiversity, DNA barcoding, Hymenoptera, Sub-Arctic, Parasitoids, Canada

## Abstract

**Background:**

Insect diversity typically declines with increasing latitude, but previous studies have shown conflicting latitude-richness gradients for some hymenopteran parasitoids. However, historical estimates of insect diversity and species richness can be difficult to confirm or compare, because they may be based upon dissimilar methods. As a proxy for species identification, we used DNA barcoding to identify molecular operational taxonomic units (MOTUs) for 7870 Hymenoptera specimens collected near Churchill, Manitoba, from 2004 through 2010.

**Results:**

We resolved 1630 MOTUs for this collection, of which 75% (1228) were ichneumonoids (Ichneumonidae + Braconidae) and 91% (1484) were parasitoids. We estimate the total number of Hymenoptera MOTUs in this region at 2624-2840.

**Conclusions:**

The diversity of parasitoids in this sub-Arctic environment implies a high diversity of potential host species throughout the same range. We discuss these results in the contexts of resolving interspecific interactions that may include cryptic species, and developing reproducible methods to estimate and compare species richness across sites and between surveys, especially when morphological specialists are not available to identify every specimen.

## Background

The region surrounding the town of Churchill, Manitoba, lies at the intersection of the tundra and boreal forest biomes, making it an ideal site for investigating ecotone biodiversity. Previous studies of hymenopteran subtaxa [1,2] and aquatic insects [3,4] have increased previous species richness estimates for this region by integrating morphological and molecular methods for species delimitation.

Members of the order Hymenoptera include pollinators, other herbivores, parasitoids, hyperparasitoids, predators, cleptoparasites, inquilines, and omnivores [5], so hymenopteran species play multiple and complex roles in terrestrial food webs. However, accurate characterization of trophic interactions is impossible without a better understanding of community biodiversity. Indeed, a historic underestimation of biodiversity has undoubtedly hampered our understanding of Arctic food webs [6], while integrative approaches reveal previously unsuspected cryptic diversity within parasitoid-host systems [7].

By most measures, biodiversity gradients generally show higher species richness at lower latitudes, but parasitoid Hymenoptera have been a commonly noted exception in some earlier literature, at least in comparisons of tropical vs. temperate regions [8,9]. Other studies suggest that this conclusion is incorrect, arguing that it is an artifact created by errors of scale [10,11], overrepresentation of some study locations [12], inconspicuousness or low abundance of some taxa [13], incomplete or inconsistent sampling methods [12,14,15], and even a latitudinal bias in incorrect taxonomy [16].

The general pattern of reduced biodiversity at high latitudes has been attributed to harsh climate [17,18], slower evolutionary rates due to long generation times [17], loss of populations via drift or inbreeding depression [17], and geographic/geological barriers to colonization [19]. At the same time, other studies have found higher-than-expected diversity among Arctic and sub-Arctic Hymenoptera, both at our study site and in similar environments. For example, a recent study of the boreal and Arctic Microgastrinae (Braconidae) increased by 50% the estimate of species richness for this subfamily in Canada and Alaska [20].

In the context of sub-Arctic insect ecology, “expected” biodiversity is difficult to define. For example, anthropogenic climate change may disproportionately affect high-latitude regions [2,21], making older diversity estimates obsolete. Molecular methods for quantifying species richness typically exceed estimates made via morphology alone, because molecular methods both reveal cryptic species and permit inclusion of specimens that would otherwise remain unidentified [1,22]. Comparisons among regions should be testable via independent genetic and morphological evidence, but the large scale of most ecological surveys make it impractical either to examine visible characteristics or to sequence multiple genes from every specimen [22].

We here report both the observed diversity and the estimated richness for both parasitoid and non-parasitoid Hymenoptera of the Churchill region, using abundance-based data from a comprehensive collection made between 2004 and 2010. We also assessed similarity of assemblages among local collection sites, using incidence-based data from a subset of specimens collected during the 2010 season. Our species counts and estimates are based on DNA barcodes [23], which we assigned to molecular operational taxonomic units (MOTUs) [24]. We used these data to construct rarefaction curves, investigate diversity of subtaxa, and estimate species richness for Hymenoptera of the Churchill region. This approach has several advantages. First, it provides a proxy for species-level determinations, which were not available for the entire collection (and rarely are available for collections of this size). Second, it reveals suspected cryptic species that can be further studied and eventually described if their existence is supported via integrative methods [25,26]. Third, this approach can be standardized, which will make it easier for future researchers to compare results among similar regional diversity studies.

We discuss our results in the context of overall hymenopteran diversity, the composition of different feeding guilds, and the potential of our approach to inform future studies of Arctic and sub-Arctic insect communities.

## Results

### MOTU counts

For the overall Churchill collection (N = 7870), MOTU counts calculated with jMOTU ranged from a high of 1898 (cutoff divergence = 1%) to a low of 1221 (cutoff divergence = 3%). For the Churchill 2010 subcollection, MOTU counts ranged from a high of 798 (1% cutoff) to a low of 660 (3% cutoff). The overall Churchill collection and the 2010 subcollection are described in more detail in the Methods section. For brevity, we report remaining results for only the 2% cutoff unless otherwise specified.

In the overall collection, we resolved 1630 MOTUs at the 2% cutoff level. The families represented by the most individuals – Ichneumonidae (n = 4797) and Braconidae (n = 1367) – were also the most diverse. We resolved 915 MOTUs for Ichneumonidae and 313 MOTUs for Braconidae. The dominance of these two families was also evident in the 2010 subcollection, for which we resolved 722 MOTUs at the 2% cutoff level. This subcollection included 1460 ichneumonids (461 MOTUs) and 288 braconids (127 MOTUs). Ichneumonoids made up 78% of the specimens in the full collection and 83% of the 2010 subcollection. Ichneumonoid MOTUs also made up 75% (= 228/1630) of overall MOTUs and 81% (= 588/722) of the subcollection MOTUs.

Along with the Ichneumonoidea, other parasitoids dominated the collection. The superfamily Chalcidoidea accounted for 321 individuals (132 MOTUs) in the overall collection and 172 individuals (42 MOTUs) in the 2010 subcollection. The most diverse taxon outside the Ichneumonoidea and Chalcidoidea was another parasitoid family – the family Diapriidae (Diaprioidea), with 163 individuals (83 MOTUs) in the overall collection and 80 individuals (45 MOTUs) in the 2010 subcollection.

Among non-parasitoids, the most numerous and diverse family was the Tenthredinidae, with 479 individuals (72 MOTUs) in the overall collection and 63 individuals (23 MOTUs) in the 2010 subcollection. This family accounted for all of the Symphyta in the Churchill collection, except for seven individuals of a single species (*Trichiosoma triangulum*) of the family Cimbicidae (Tenthredinoidea), which were present in the overall collection.

Full specimen and MOTU counts for all represented families and/or superfamilies of Hymenoptera are summarized in Table 1.

**Table 1:** Specimen counts, feeding ecology, and number of MOTU (2% divergence) for overall Churchill collection and 2010 subset

**Superfamily**	**Family**	**Feeding ecology**	**N (all)**	**MOTU (all)**	**N (2010)**	**MOTU (2010)**
**Apoidea**	Andrenidae	Herbivore	5	3	0	0
	Apidae	Herbivore	82	8	0	0
	Crabronidae	Predator	7	6	0	0
	Halictidae	Herbivore	19	3	1	1
	Megachilidae	Herbivore	15	6	0	0
**Ceraphronoidea**	Megaspilidae	Parasitoid	2	1	1	1
**Chalcidoidea**	–	Parasitoid	321	132	172	42
**Chrysidoidea**	Chrysididae	Cleptoparasite	4	4	0	0
	Dryinidae	Parasitoid	18	3	9	2
**Cynipoidea**	–	Mixed/other	36	28	10	9
**Diaprioidea**	Diapriidae	Parasitoid	163	83	80	45
**Ichneumonoidea**	Braconidae	Parasitoid	1367	313	288	127
	Ichneumonidae	Parasitoid	4797	915	1460	461
**Platygastroidea**	Platygastridae	Parasitoid	40	29	5	3
**Proctotrupoidea**	Proctotrupidae	Parasitoid	23	8	19	6
**Tenthredinoidea**	Cimbicidae	Herbivore	7	1	0	0
	Tenthredinidae	Herbivore	479	72	63	23
**Vespoidea *s.l.***	Formicidae	Mixed/other	373	7	2	1
	Pompilidae	Predator	1	1	1	1
	Vespidae	Predator	111	7	0	0
**TOTAL**			**7870**	**1630**	**2111**	**722**

### Accumulation/rarefaction curves and richness estimates

**Figure 1:**
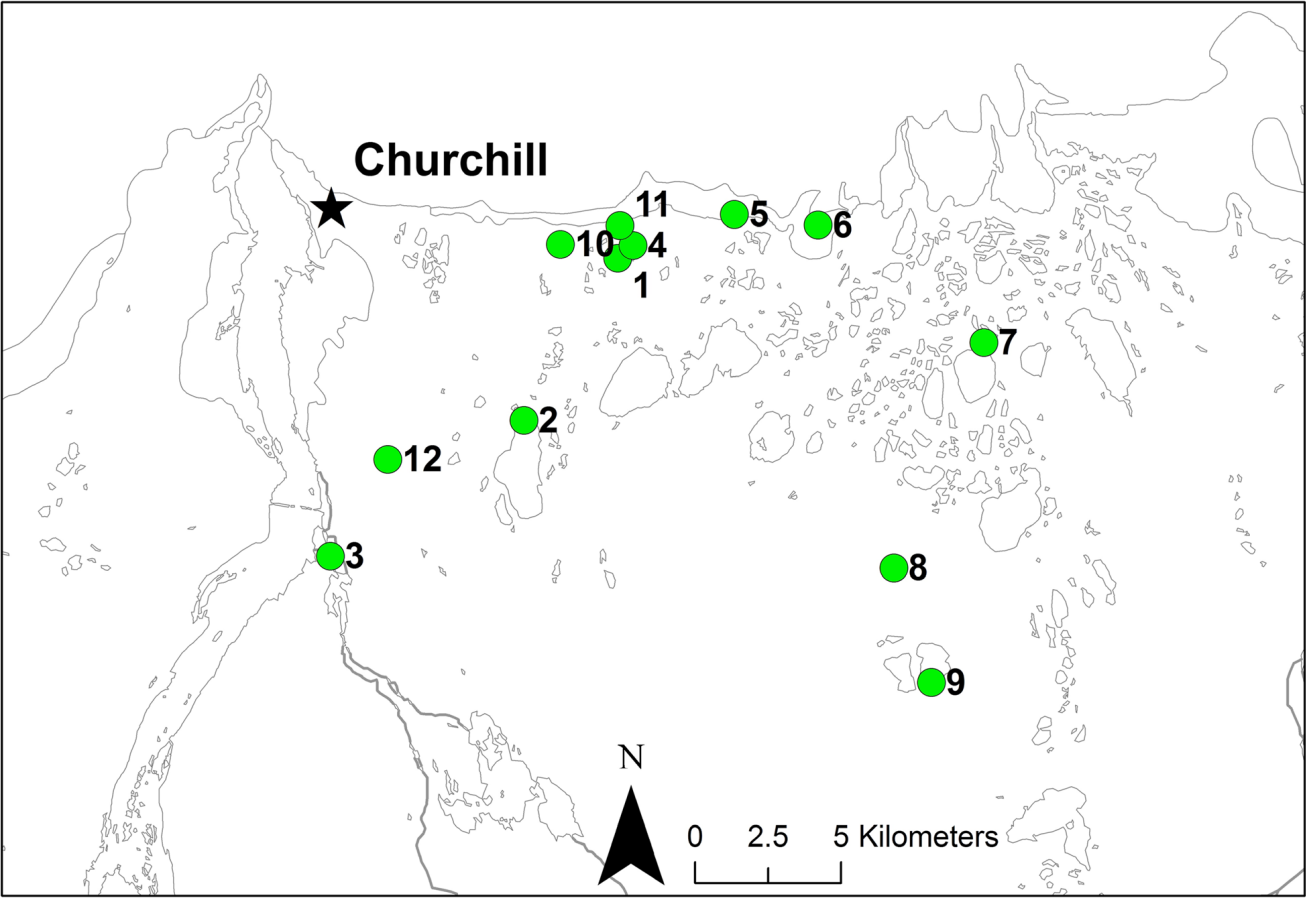
**Abundance-based rarefaction curves for entire Churchill Hymenoptera 2004-2010 collection (N=7870), for MOTU cutoff values of 1% through 3%.** Dotted curves represent +95% CI (for 1% cutoff) and -95% CI (for 3% cutoff).

**Figure 2:**
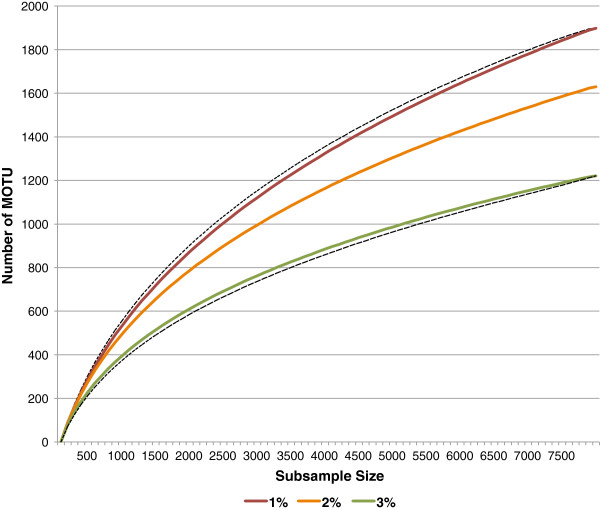
**Incidence-based rarefaction curves for Churchill Hymenoptera 2010 subcollection (N=2111), for MOTU cutoff values of 1% through 3%.** Dotted curves represent +95% CI (for 1% cutoff) and -95% CI (for 3% cutoff).

Rarefaction curves for the complete Churchill collection and the 2010 subcollection are shown in Figures 1 and 2. Neither set of curves approaches an asymptote at the point where all individuals (Figure 1) or samples (Figure 2) are included.

For the overall collection, the Chao 1 richness estimate [27] was 2624, with a 95% CI range of 2446 through 2840. For the 2010 subcollection, the Chao 2 richness estimate [28] was 1384, with a 95% CI range of 1233 through 1578. Additional results for cutoff ranges from 1% through 3% are shown in Table 2.

**Table 2:** Estimated species richness of Churchill Hymenoptera, for MOTU cutoff values of 1%- through 3%

**Cutoff**	**MOTU**	**Chao 1 richness**	**Chao 1 95% CI**	**Chao 1 95% CI**
			**lower bound**	**upper bound**
1%	1898	3187	2977	3438
2%	1630	2624	2446	2840
3%	1221	1913	1770	2093
**Cutoff**	**MOTU**	**Chao 2 richness**	**Chao 2 95% CI**	**Chao 2 95% CI**
			**lower bound**	**upper bound**
1%	798	1611	1436	1833
2%	722	1384	1233	1578
3%	660	1194	1067	1362

Upper table: Entire Churchill Hymenoptera collection (abundance-based, Chao 1). Lower table: Churchill 2010 subcollection (incidence-based, Chao 2).

### Species similarity among sites

Sobs [29,30] for each of the 12 sites in the 2010 subcollection ranged from a low of 43 (site 8, 23 km SE) to a high of 221 (site 2, 10 km SE). The largest estimated shared species value was 413, between sites 5 and 12 (12 km ESE and 9 km S), and the smallest was one, between sites 3 and 6 (10 km SE and 14 km E).

**Figure 3:**
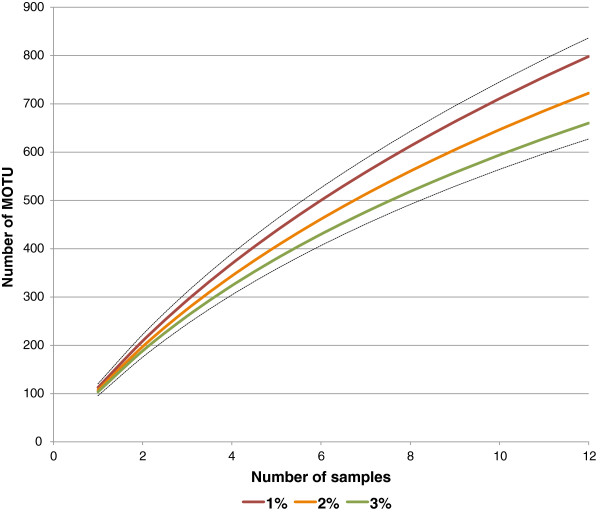
**Chao-Sørensen-Est similarity index for each pair of samples in the Churchill 2010 subcollection.** The trendline is not significant (p = 0.42, r = 0.10, df = 64).

The highest value of the Chao-Sørensen-Est abundance-based similarity index [31] between any two sites was 0.867, between sites 8 and 9 (23 km SE and 26 km SE, ~ 5 km apart). The lowest similarity index was 0.020, between sites 6 and 10 (16 km E and 7 km ESE, ~ 9 km apart). There was no significant correlation between similarity index and the distance between sites (p = 0.42, r = 0.10, df = 64; Figure 3).

Full results (2% cutoff) for shared species and similarity are included in Additional file 1. Because Sobs for each site includes MOTUs shared with other sites, the Sobs values for the 12 sites do not sum to the total Sobs for the 2010 subcollection.

### Proportional contribution of taxa and feeding guilds

**Figure 4:**
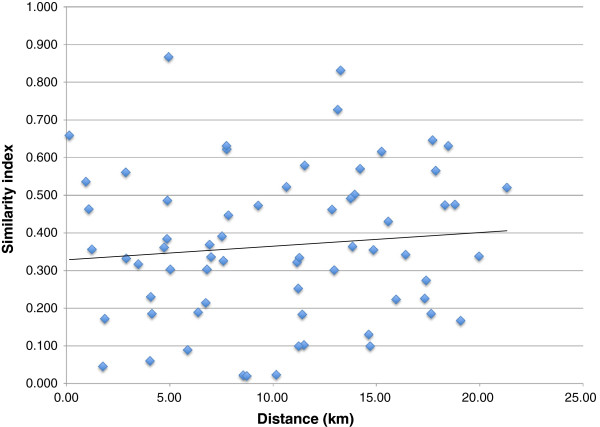
Relative contributions by taxon to MOTU diversity in the complete Churchill Hymenoptera collection.

**Figure 5:**
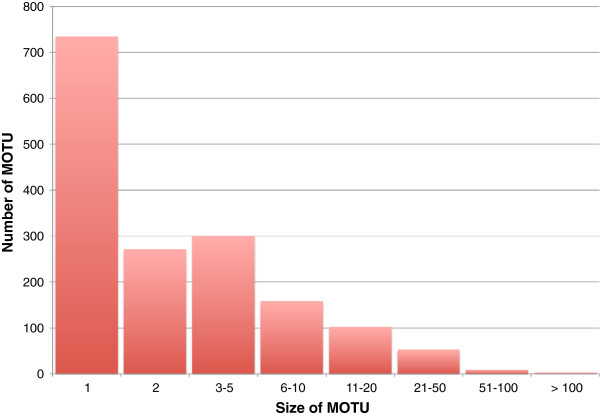
Relative contributions by feeding guild to MOTU diversity in the complete Churchill Hymenoptera collection.

Relative contributions to overall MOTU diversity within the overall Churchill collection are shown in Figure 4 (by taxon) and Figure 5 (by feeding guild). These proportions remained similar throughout the 1-3% sequence divergence cutoff ranges for MOTU assignments. Proportions for the Churchill 2010 subcollection were similar to those for the overall collection (data not shown).

## Discussion

### Species identification and delimitation

**Figure 6:**
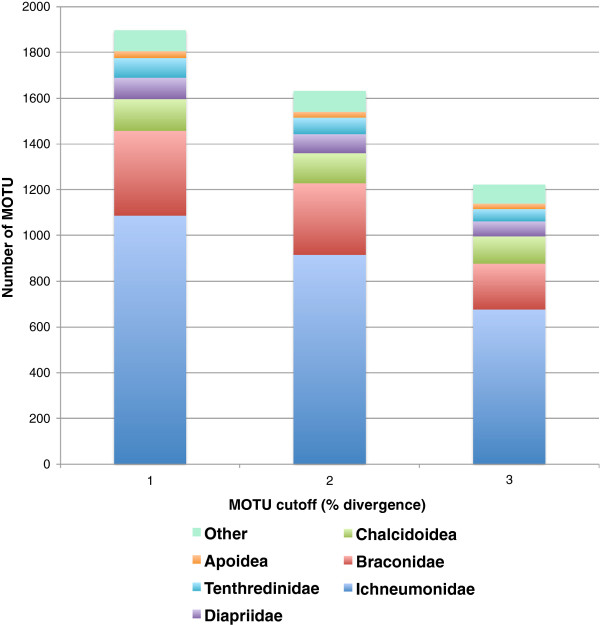
**Distribution of MOTU sizes (= individuals per MOTU) in the complete Churchill 2004-2010 collection.** X-axis not to scale, in order to emphasize high frequencies of singletons, doubletons, and other MOTUs with small numbers of replicates.

The 7870 specimens from the Churchill collection were assigned to 1630 MOTUs at the 2% cutoff range. On average, we identified a different species for every 4.8 individuals sampled (SD = 11.3). Most MOTU were sparsely represented, with a mode of 1 (n = 734) and a median of 2. Only 194 MOTUs included 10 or more individuals, and only three of these included 100 or more (Figure 6). For the Churchill 2010 subcollection, there were 722 MOTUs, and the mean number of individuals per MOTU was 2.9 (SD = 4.5, mode = median = 1, maximum = 55, but pre-sorting of this subcollection limited the number of replicates per MOTU). Singleton MOTUs made up a similar proportion of the 2010 subcollection as they did of the parent collection (362 MOTUs = 50% of the 2010 subcollection; 734 MOTUs = 45% of the parent collection).

Based on number of MOTUs, the most diverse families in the overall Churchill collection were the Ichneumonidae (915 MOTUs), Braconidae (313 MOTUs), Diapriidae (83 MOTUs) and Tenthredinidae (72 MOTUs). All of these families except the Diapriidae included some specimens that had been previously identified below the family level. We also resolved 132 MOTUs among the Chalcidoidea, which were not determined to family. Each of the other taxonomic groups in this study, including the entire superfamily Cynipoidea, yielded fewer than 30 MOTUs.

As determined by traditional morphological taxonomy, the 4787 specimens of Ichneumonidae in the overall collection encompassed at least 13 subfamilies, 34 genera, and 49 species. However, 1861 individuals (38.9%) carried no identification below the family level. At the 2% cutoff level, 915 MOTUs were resolved for this family; if this cutoff level provides a good approximation to interspecific divergence in Ichneumonidae, then morphological examination previously resolved only 5.4% of the diversity present. Similarly, for 1367 specimens of Braconidae, 8.6% had no identification below the family level and the number of resolved species was only 40.9% of the MOTU total. For 479 Tenthredinidae specimens, 23.2% had no identification below the family level, and the resolved species count was 25.0% of the MOTU total.

These values should be considered approximations, for several reasons. First, no single MOTU cutoff level can definitively delimit all species; the 2% cutoff level for MOTU definition is known to be insufficiently stringent for some hymenopteran subtaxa and too stringent for others [1,2,32,33]. Second, even the better-identified families in this collection have not to date received the same amount of scrutiny by morphological taxonomists, and the most completely determined taxa as of May 2012 (bees, vespid wasps, and microgastrine braconids) made up only a small proportion of the collection.

The second issue – unequal attention given to different taxa – is not a trivial one, because full morphological species identification requires considerable effort and expertise, and identifying a diverse collection requires a diverse assemblage of specialists. The low rate of morphological specimen identification says little about the ability of taxonomists to identify a specimen in hand, but much about the difficulty of placing every specimen from a large survey into the hands of the appropriate specialist [23,34]. Specimens from this study may receive more attention from specialists in the future, and the availability of DNA barcodes will make it easier for taxonomists to delimit and perhaps even describe species in this collection via integrative methods [25,35].

### Rarefaction and richness estimates

Rarefaction/accumulation curves show no clear approach to asymptotes for either the complete Churchill collection (abundance-based) or the 2010 subcollection (incidence-based over 12 sites) The Chao 1 richness estimate at 2% cutoff is 2624 MOTUs, with a 95% CI of 2446 through 2840. By this estimate, the 1630 MOTUs defined from our collection cover approximately 62% ± 5% of total richness. Since this estimate was based on MOTUs rather than on morphological determinations, it is independent of the amount of attention each subtaxon received from a morphological taxonomist. It should still be considered exploratory, because we did not sequence every one of the many thousands of individual specimens originally collected, and because the overall collection was not controlled for variation in sampling effort at different locations throughout the collection range.

Abundance-based measures may also be biased by over- or under-representation of some species independent of site selection or sampling effort. For example, our collection contained social insects (ants, bumble bees, and vespines) that can be oversampled when traps are set near nests or other sites of high colony activity. As individual specimens, social Hymenoptera were among the most abundant members of the collection, but also among the least diverse, and this was verified by both sequence data and morphological examination. Ants comprised 7 MOTUs, including *Camponotus herculaneus* (n = 27), two apparently distinct members of the *Formica fusca* complex (n_1_ = 45 and n_2_ = 282,) one species of *Myrmica* (*Myrmica alaskensis*, n = 9,) and up to three members of the genus *Leptothorax* (n_1_ = 1, n_2_ = 4, n_3_ = 5). Similarly, the collection included seven identified species of *Bombus* whose sample sizes ranged from a minimum of two (*B. flavifrons*) to a maximum of 44 (*B. sylvicola*), and which formed eight MOTUs (jMOTU split *B. mixtus* and *B. sylvicola* each into two MOTUs, and pooled *B. frigidus* and *B. jonellus* into one MOTU). Three species of Vespinae were also present, with sample sizes ranging from eight (*Vespula intermedia*) to 87 (*Dolichovespula albida*). Vespine wasps were assigned to three MOTUs that were congruent with morphological identifications.

### Similarity among sites

For the 2010 subcollection, values of the Chao-Sørensen-Est abundance-based similarity index were highly variable among different site pairs, ranging from 0.020 to 0.867. However, these values were not significantly correlated with the linear distances between site pairs (Figure 2).

Almost all specimens in the Hymenoptera collection were of winged insects or their larvae, and we expect these species to disperse widely among sites. The only exceptions were worker ants and female Dryinidae; however, since ant reproductives and male dryinids are winged, these species can still disperse via flight. The wide range of between-site similarities could be related to microhabitat differences, variation among sampling methods, or sampling at different times throughout the season. For example, even in the short sub-Arctic summer, the Trichoptera of the Churchill region show some seasonal variation in species presence and abundance [4], and this may be true of Hymenoptera as well. The Chao-Sørensen-Est similarity model should mitigate some of these effects, since it includes an estimate of unseen species and also accounts for sample size difference (the latter indirectly, via species/MOTU counts at each pair of sites). Whether or not methodological or microhabitat differences affected incidence or abundance measures of some MOTUs, the 2010 subcollection showed no evidence for distance-dependent substructure across the study area.

### Feeding ecology and the importance of parasitoids

The hymenopteran fauna of the Churchill region is dominated by parasitoids, and the parasitoid fauna of Churchill is dominated by the Ichneumonoidea (Ichneumonidae + Braconidae). We observed this pattern not only in the overall collection, but also in the 2010 subcollection, in which specimens were deliberately selected for inclusion by a method designed to maximize taxonomic breadth.

Worldwide, the Ichneumonidae and Braconidae are thought to be the first and second most diverse families of Hymenoptera [5,36], so this result is not unexpected in any regional survey of the order. Only about 12% of identified parasitoid MOTUs were non-ichneumonoids, but these represented at least six different hymenopteran superfamilies, including members of the families Diapriidae (Diaprioidea), Dryinidae (Chrysidoidea), Megaspilidae (Ceraphronoidea), Platygastridae (Platygastroidea), Proctotrupidae (Proctotrupoidea), plus multiple families of Chalcidoidea.

Still, parasitoids cannot reproduce without hosts, so the diversity of parasitoids in this sub-Arctic location implies a high level of host diversity. Many members of the superfamily Ichneumonoidea are parasitoids of immature Lepidoptera, but they also parasitize hosts from other holometabolous orders, nymphal Hemimetabola, and arachnids [5,37,38]. At least four ichneumonid subfamilies (Campopleginae, Ctenopelmatinae, Tersilochinae, and Tryphoninae) target sawflies, and others (e.g. Mesochorinae) include hyperparasitoids. Non-ichneumonoid parasitoids can also exploit a wide range of hosts, including Diptera (by Diapriidae), Hemiptera (by Dryinidae), and the variety of hosts that are susceptible to attack by Chalcidoidea [5,37]. A more detailed list of Churchill parasitoid subtaxa and their presumed hosts is included in Additional file 1.

Parallel studies of the Churchill fauna reveal no shortage of potential hosts. Our Hymenoptera collection includes more than 70 presumed species of tenthredinoid sawflies. The comprehensive Churchill terrestrial arthropod collection includes approximately 1800 species of Diptera, 315 of Lepidoptera, 300 of Coleoptera, 90 of Hemiptera, and 200 of Araneae [S.J. Adamowicz et al. pers. comm.]. These numbers are probably conservative, since they are based on observed MOTU or species richness rather than on extrapolated richness. If true species richness of these other orders is similar to that of Hymenoptera (e.g. approximately one-third of the true richness is not accounted for in the collection), then we would expect that more than 4000 species from these groups are potential hosts for hymenopteran parasitoids in the Churchill region. Details of host-parasitoid interactions are far beyond the scope of this survey, so we cannot tell whether some parasitoids attack multiple hosts, or whether some hosts are attacked by multiple parasitoids. However, DNA barcode data can provide the earliest clues to the presence of cryptic host-parasitoid interactions that can be targeted for future taxonomic and ecological study [39,40].

Non-parasitoids in this collection were dominated by obligate herbivores, including bees (Andrenidae + Apidae + Halictidae + Megachilidae, 20 MOTUs) and sawflies (Tenthredinidae, 73 MOTUs). Predators were much less diverse (Crabronidae, six MOTUs, Pompilidae, one MOTU, and Vespidae, seven MOTUs). Other aculeates included Chrysididae (four MOTUs) and Formicidae (seven MOTUs). The chrysidids – all members of the cleptoparasitic subfamily Chrysidinae – are likely to invade nests of host taxa that were represented in this collection [41]. One chrysidine, *Omalus aeneus*, is a known cleptoparasite in nests of *Pemphredon* spp*.* (Crabronidae), a genus represented by two individuals in the overall collection. The other chrysidines were not identified to species, but some Nearctic chrysidines are specialized cleptoparasites of the cavity nests of eumenine vespids [41]. The overall collection contained three determined species of cavity-nesting Eumeninae: *Ancistrocerus albophaleratus, A. waldenii*, and *Euodynerus leucomelas*[42].

### Geography and climate

Some previous surveys of regional ichneumonid fauna showed an unusual pattern – a higher diversity of Ichneumonidae (but not necessarily other parasitoid taxa) in some temperate regions than in the tropics [8,43]. However, this conclusion has been challenged [13,44], because in practice, it is difficult to design and implement a sampling protocol free of methodological bias. As a result, spurious patterns of diversity can be traced to over- or under-sampling of some subtaxa or habitats that share characteristics other than latitude [12,13,45]. Some of these factors are additionally complicated by uncertain taxonomy. For example, molecular evidence may reveal that some “generalist” parasitoid species are actually made up of multiple cryptic species with more narrow host specializations than previously recognized [15,39,40].

These issues make it difficult to define *a priori* expectations of parasitoid diversity in a sub-Arctic environment, and to compare it with diversity at other latitudes. For example, the earliest descriptions of anomalous latitudinal gradients in parasitoid diversity pre-date the routine use of both large-scale trapping [14,46] and molecular methods [47,48] in biodiversity research. The MOTU approach is not always sufficient for species delimitation, and is not sufficient to describe new species, but it has the advantage of being quantitative, reproducible, and feasible even when morphological determinations are impractical [24]. As such, barcode-based diversity studies can reveal large-scale geographic or ecological patterns of cryptic diversity, and can also identify specific taxa in need of further study by specialist taxonomists or conservation biologists.

## Conclusions

The temptation to describe a regional fauna as unexpectedly diverse is often high, especially in surveys of extreme environments. The resolution of more than 1600 species of Hymenoptera in the Churchill region provides just such a case, because of both the harshness of this environment and the dominance of parasitoids. The success of parasitoids is in turn a clue to the previously unexplored diversity of their hosts, especially other Holometabola.

Full species delimitation and description require more detail than DNA barcoding alone can provide; these are questions best answered by integrative methods that incorporate morphology, ecology, and genetics. However, in environments like that of sub-Arctic Canada – environments disproportionately threatened by climate change – taxonomists and ecologists have the twin additional challenges of limited time and limited resources. It is currently difficult to compare the diversity of this region to either other regions or its own past, because of the varied methods used in historical studies. DNA barcoding, combined with permanent curation of the barcoded specimens, provides an avenue for rapid estimation of species richness, as well as an easily standardized method for designing and comparing future studies and prioritizing critical research.

## Methods

### Specimen collection, identification, and curation

Hymenoptera specimens were part of a larger arthropod collection made in the Churchill area between July 2004 and August 2010, inclusive. The majority of Hymenoptera specimens were collected via malaise trapping and sweep netting. Latitude of collection sites ranged from 58.630° through 58.755°, and longitude from -93.819° through -93.998°.

We randomly sampled approximately 20% of the entire Churchill Hymenoptera collection for barcoding (but see description below for the more detailed selection protocol for specimens collected in 2010.) Specimens were sorted to the order level at the Biodiversity Institute of Ontario (BIO, Guelph, ON, Canada) and at the Canadian National Collection of Insects, Arachnids, and Nematodes (CNC, Ottawa, ON, Canada). A total of 9562 specimens were submitted to the Canadian Centre for DNA Barcoding (CCDB) laboratory at BIO for DNA isolation, amplification, and sequencing. Voucher specimens were retained at these locations after tissue was sampled for DNA analysis. Collection data, sequence data, and GenBank accession numbers for these specimens are available through the Barcode of Life Database (BOLD, http://www.boldsystems.org/), in the dataset DATASET-HYMCHUR1 (doi:http://dx.doi.org/10.5883/DATASET-HYMCHUR1). More precise taxonomic determinations have been added for some specimens since their initial identification, and further taxonomic detail will be added to the BOLD dataset as work progresses after publication. These updates will be entered into BOLD and will become accessible through this dataset. A summary of specimen records and data, including GenBank accession numbers, is included in Additional file 1.

### DNA extraction, amplification and sequencing

DNA was extracted from a single leg or abdomen of each adult specimen, or a tissue sample from each immature specimen, according to the methods of Ivanova *et al.*[49]. Standardized primer sets [50,51] were used to amplify and sequence the 658-bp barcode region [23]. When amplification or sequencing failed, DNA was re-amplified and re-sequenced using primers that amplify smaller overlapping fragments and yield shorter sequences, but are still useful for specimen identification and sequence analysis [52,53]. A list of the primers used for barcoding of these specimens is provided in Additional file 1.

### Dataset construction and verification

We constructed our primary dataset from the complete Churchill collection of Hymenoptera specimens that met the following criteria: (a) Collected between 2004 and 2010, inclusive; (b) Sequence ≥ 200 bp; (c) Sequence check against BOLD ID engine consistent with existing ID or image; (d) No stop codons in sequence; (e) Fewer than 10 ambiguous bases for sequences of length ≥ 350 bp, and fewer than five ambiguities for shorter sequences; and (f) Sequence not previously published. Criteria (a) through (e) were used to exclude pseudogenes, contaminated DNA samples, and low-quality sequences. Of the 9562 specimens originally available, 7870 (82.3%) fit all criteria for inclusion. At the time of data analysis, only 1402 of the 7870 specimens (18%) had been identified to the level of species, provisional species, or species complex via morphology. So, for consistency, we subsequently refer to specimens only by family (superfamily for the Cynipoidea and Chalcidoidea). For 7788 specimens (99%), at least one image is present in BOLD. There were two years of particularly intensive study for Hymenoptera (2007 and 2010), while the full dataset included four specimens collected in 2004, 88 in 2005, 887 in 2006, 3464 in 2007, 432 in 2008, 457 in 2009, and 2538 in 2010. The complete 2004-2010 dataset was used to estimate species count and abundance-based species richness for Hymenoptera of the Churchill region.

**Figure 7:**
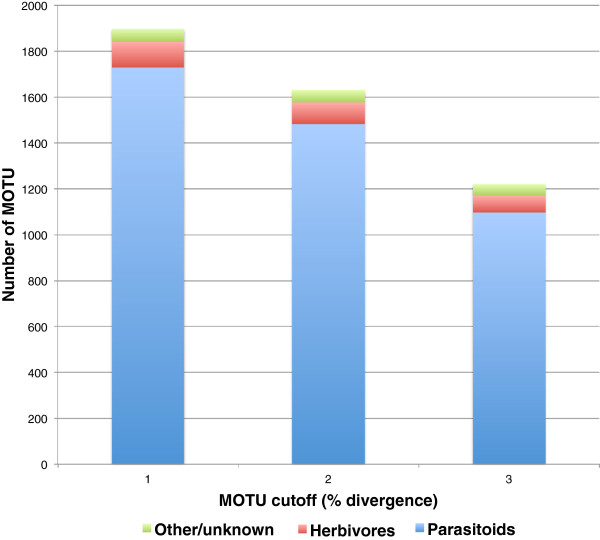
**Map of Churchill, Manitoba, region, showing the 12 sites sampled for incidence-based analyses in the 2010 subcollection and their locations with respect to the town of Churchill.** Further site details are available in Additional file 1.

We also assembled a second dataset (a subset of the first), designed to further investigate similarity of species assemblages, and to estimate species richness via an incidence-based method. These sites were chosen because they yielded collections of comparable sizes, and all collections from these sites were sorted by the same researcher (MTM). The second dataset included 2111 specimens that were all collected between 1 July and 15 August 2010, from 12 sites that each yielded 50 or more specimens (Figure 7). These sites span the range of habitats at Churchill, including fen, bog, boreal forest, tundra, and exposed Hudson Bay shoreline (Additional file 1). Each one of these sites constituted one sample for the purpose of incidence-based analyses.

Of the 2111 specimens, 1257 were collected from malaise traps, 822 by sweep netting, 26 from pan traps, and six by other methods. Specimens were sorted by collection event and morphospecies, with the goal of capturing maximum diversity while minimizing redundancy. Beginning with the largest specimens from each event, we selected 10 specimens per known parasitoid morphospecies and 5 specimens per other morphospecies for molecular analysis. For some large and distinctive social insects (e.g. Vespinae), fewer than 5 individuals were included.

### Species/MOTU delimitation

Because many individuals were not morphologically determined, we assigned each specimen to a MOTU using the program jMOTU [54]. To reduce computational requirements, we divided our overall data into subsets of < 1400 sequences. Subsets were delimited by taxon, though not always at the same level (e.g. all chalcidoids were pooled in one subset, while the Ichneumonidae were split into six subsets based on their cluster positions in a preliminary BOLD NJ tree). This method of partitioning the dataset prevented accidental duplication of MOTU assignments across separate jMOTU runs. All jMOTU runs included MOTU cutoff definitions between 1 bp and 66 bp, covering a range of 0.15% to 10% divergence with respect to the full-length (658 bp) barcode. We used a low BLAST identity filter of 95%, and a required sequence alignment overlap of 60% of minimum sequence length.

For rarefaction/accumulation curves and estimates of species richness, we constructed preliminary data files from a range of outputs corresponding to different cutoff percentages that were defined in relation to the maximum length of the barcode sequences (658 bp). We selected cutoffs of 1% (7 bp), 2% (13 bp), and 3% (20 bp) for further analyses, based on prior evidence that they span the range of divergences most useful for delimiting hymenopteran species [1,2,32,33]. Also, results in this range of cutoff values were relatively unaffected by fragment length differences, permitting the inclusion of a wider range of sequences in the dataset. Outputs from jMOTU at these cutoff levels, including representative demonstrations of sequence length effects, are included in Additional file 1.

### Rarefaction curves, species richness, and shared species

For the full Churchill collection, we constructed abundance-based rarefaction curves for each cutoff level. For rarefaction of the full collection, we selected subsample sizes ranging from 100 to 7800 in intervals of 100. The 95% confidence intervals for these curves were based on 1.96 * standard deviation; these intervals always converge on the actual count of individuals when maximum N is reached, because at that point there is no replication (SD = 0).

For the Churchill 2010 subcollection, we constructed sample-based rarefaction curves based on the observed-richness function Sobs (Mao Tau) [55], computed for one through 12 subcollection sites. For these curves, 95% confidence intervals were calculated under the assumption that the entire dataset represented a sample from a larger pool; these intervals do not converge on the actual count at maximum N.

We estimated species richness for the complete collection using the abundance-based Chao 1 estimator [27] and for the 2010 subcollection using the incidence-based Chao 2 estimator [28]. For each pair of sites in the 2010 subcollection, we calculated observed shared species (Sobs), and estimated the actual number of shared species using Chao’s coverage-based estimator [29]. We also calculated the Chao-Sørensen-Est similarity index for each pair. This is an abundance-based estimate of the probability that if one individual is chosen from each of two sites, both belong to species that are shared between the sites [31]. This model also accounts for the probability of some species remaining unsampled when actually present.

All rarefactions, richness estimates, and shared species parameters were generated using EstimateS 8.2 [30,55], except for the abundance-based rarefaction curves for the complete Churchill collection. We used default EstimateS 8.2. settings of 50 randomizations of input order and 200 bootstrap replicates. Abundance-based curves for the complete collection were generated with the Rarefaction Calculator [56] because EstimateS outputs do not include this level of detail for a collection defined as a single sample.

### Feeding ecology

We defined as parasitoids all members of the superfamilies Ichneumonoidea, Ceraphronoidea, Chalcidoidea, Diaprioidea, Platygastroidea, and Proctotrupoidea, plus the family Dryinidae (Chrysidoidea). We defined as herbivores all bees (Andrenidae, Apidae, Halictidae, and Megachilidae) and sawflies (Cimbicidae and Tenthredinidae). Groups with other feeding behaviours formed a much smaller proportion of the dataset, and were defined as mixed/unknown/other. These groups included predators and similar provisioners (Crabronidae, Pompilidae, Vespidae), omnivores (Formicidae), and cleptoparasites (Chrysididae). The superfamily Cynipoidea was also considered an ecologically mixed taxon, as it contains both Cynipidae (plant gall formers) and Figitidae (parasitoids), but too few were determined to family level to enable reliable assignment of cynipoids to feeding guilds. Cynipoids are included in overall diversity measures, but in discussions of feeding ecology they are counted as mixed/unknown/other.

## Abbreviations

BOLD: Barcode of Life Data Systems (http://www.boldsystems.org); MOTU: Molecular operational taxonomic unit.

## Competing interests

The authors declare that they have no competing interests.

## Authors’ contributions

JKS, JFT, SA, and MAS wrote the manuscript. JKS and MAS managed the BOLD projects and analyzed the sequence data. JFT identified the majority of determined specimens and coordinated curation efforts at the CNC. HG, TW, JH, CS, MB, MAS, and JKS identified additional specimens. MM organized and prepared the 2010 subcollection. MAS and TW contributed supplementary graphics. SA and PDNH developed the study and organized the fieldwork. PDNH, SA, and MAS provided institutional support and scientific consultation throughout the project. PDNH led the grant applications that funded the work. All authors read and approved the final manuscript.

## Authors’ information

Julie K Stahlhut and José Fernández-Triana are joint first authors.

## Supplementary Material

Additional file 1 **Supplementary lists and tables for Churchill Hymenoptera collection.** This is an Excel file (.xlsx) with multiple worksheets. Worksheet index below: **Sheet 1:** Specimen records for complete Churchill collection. **Sheet 2:** Specimen records for Churchill 2010 subcollection. **Sheet 3:** Primers used for amplification of Hymenoptera sequences at the Canadian Centre for DNA Barcoding.**Sheet 4:** jMOTU outputs for complete Churchill collection for cutoffs of 1%-3%. **Sheet 5:** Comparisons of sequence length effects on jMOTU results for cutoff values of 1-10% divergence (relative to 658 bp). **Sheet 6:** Parasitoid taxa of Churchill and their presumed hosts. **Sheet 7:** Shared species and similarity results for all pairs among the 12 sampled sites in the Churchill 2010 collection. **Sheet 8:** Descriptions of the 12 sample sites in the Churchill 2010 collection. Click here for file
